# Neurofilament distribution in the superior labrum and the long head of the biceps tendon

**DOI:** 10.1186/s13018-017-0686-9

**Published:** 2017-11-22

**Authors:** Sandra Boesmueller, Antal Nógrádi, Patrick Heimel, Christian Albrecht, Sylvia Nürnberger, Heinz Redl, Christian Fialka, Rainer Mittermayr

**Affiliations:** 1 AUVA Trauma Center Meidling, Kundratstraße 37, 1120 Vienna, Austria; 2 Ludwig Boltzmann Institute for Experimental and Clinical Traumatology, Donaueschingenstraße 13, 1200 Vienna, Austria; 3 Department of Anatomy, Histology and Embryology, University of Szeged, Kossuth L. sgt 40, Szeged, 6724 Hungary; 4 Bernhard Gottlieb University Clinic of Dentistry, Sensengasse 2A, 1090 Vienna, Austria; 5 Department of Trauma Surgery, Medical University of Vienna, Währinger Gürtel 18-20, 1090 Vienna, Austria; 6 Medical School, Department of Trauma, Sigmund Freud University, Freudplatz 1, 1020 Vienna, Austria

**Keywords:** Neurofilament, SLAP repair, Pain, Immunohistochemistry, Histology

## Abstract

**Background:**

The postoperative course after arthroscopic superior labrum anterior to posterior (SLAP) repair using suture anchors is accompanied by a prolonged period of pain, which might be caused by constriction of nerve fibres. The purpose was to histologically investigate the distribution of neurofilament in the superior labrum and the long head of the biceps tendon (LHBT), i.e. the location of type II SLAP lesions.

**Methods:**

Ten LHBTs including the superior labrum were dissected from fresh human specimen and immunohistochemically stained against neurofilament (NF). All slides were scanned at high resolution and converted into tagged image file format, and regions of interest (ROIs) were defined as follows: ROI I—superior labrum anterior to the LHBT origin, ROI II—mid-portion of the superior labrum at the origin of the LHBT, ROI III—superior labrum posterior to the LHBT origin and ROI IV—the most proximal part of the LHBT before its attachment to the superior labrum. The entire images were automatically segmented according to the defined ROIs and measured using a programmed algorithm specifically created for this purpose. The NF-positive cells were counted, and their total size and the area of other tissue were measured separately for the different ROIs.

**Results:**

Distribution of NF-positive cells in absolute numbers revealed a clear but insignificantly higher amount in favour of ROI I, representing the superior labrum anterior to the LHBT origin. Setting ROI I at 100%, a significant difference could be seen compared to ROI III, representing the superior labrum posterior to the LHBT origin (ROI I vs. ROI III with a *p* value < 0.05).

**Conclusions:**

Summarizing, the density of neurofilament is inhomogeneously distributed throughout the superior labrum with the highest number of neurofilament in the anterior superior labrum. Thus, suture placement in type II SLAP repair could play an important role for the postoperative pain-related outcome.

## Background

Lesions of the attachment site of the long head of the biceps tendon (LHBT) at the superior labrum were first described by Codman in 1934 [1]. Andrews et al. [2] were the first to investigate this injury in detail in 1985, and Snyder et al. [3] named it superior labrum anterior to posterior (SLAP) lesion. There are four main types, ranging from a small disruption (type I) to an extensive lesion reaching from the labrum into the biceps tendon itself (type IV), and so far, even up to then, complex types are described in literature [4–6].

SLAP lesions are quite frequent and might occur in patients after a fall on the flexed and abducted arm or are caused by repetitive microtraumata in overhead athletes. Whereas there is a general consent according to the treatment of type I, type III and type IV lesions, the treatment of the type II lesion is discussed controversially throughout the orthopaedic community.

In daily routine with arthroscopically treated type II SLAP lesions, persisting pain after SLAP repair using suture anchors is often observed, which behaves disproportional to this small surgical intervention [7–10]. In contrast, the pain is obviously relieved in studies presenting the outcome after tenodesis [10, 11] or tenotomy [11, 12] of the LHBT.

The theory of a compromised blood supply of the SLAP region after SLAP repair as a potential pain generator has already been dismissed [13]. A literature review concerning other possible reasons for persistent pain brings the focus on tendinopathy of the LHBT. In their cadaver study, Hashimoto et al. [14] described large nerve endings in the LHBT as well as a Pacinian corpuscule at the boundary zone between the joint capsule and the labrum. Alpantaki et al. [15] demonstrated that the tendon of the long head of the biceps contains a large network of sensory and sympathetic nerve fibres and that the innervation is not distributed evenly throughout the tendon but rather is found predominantly near its insertion. However, all histological studies investigated either intra- or extra-articular portions of the LHBT [15–17], but none of them has extended the focus on the biceps tendon anchor comprising the most proximal part of the LHBT and the superior labrum anterior and posterior of the LHBT origin.

Thus, the purpose of this study was to histologically investigate the density and distribution of neurofilament in the LHBT as well as in the transition zone of the LHBT to the labrum, i.e. the location of type II SLAP lesions. We hypothesize that neurofilament is inhomogeneously distributed in the LHBT as well as in the anterior and posterior aspects of the superior labrum. This could be a possible explanation for the prolonged period of pain after type II SLAP repair compared to other surgical techniques like tenodesis or tenotomy.

## Methods

In cooperation with the Department of Anatomy, ten LHBTs including the superior labrum were dissected from fresh human specimen. The inclusion criterion was a macroscopically healthy tendon and superior labral region without a documented history of shoulder surgery. Exclusion criteria were macroscopically degenerated tendons (i.e. tendinosis), patients with a LHBT rupture and patients who had undergone any kind of shoulder surgery (i.e. prosthesis, rotator cuff repair and subacromial decompression). All tendons were assessed by the first (SB) and senior (RM) authors prior to histological investigation. There were five right and five left shoulders from six female and four male patients with a mean age of 74 years (range 52–89 years). All specimens were taken from body donors according to a special agreement signed by the individuals before their demise. This study has been approved by the local ethics committee (No. 139/2011).

### Immunohistochemistry

All specimens were fixed in 7.5% formaldehyde immediately after harvesting and remained for 3 days. The specimens were then embedded in paraffin, and longitudinal sections in the scapular plane of 3–4-μm thickness were performed with a rotary microtome and dried at 45 °C overnight. Afterwards, the sections were deparaffinised and rehydrated.

Several protocols of immunohistochemical stainings against neurofilament exist but have inconsistent outcome including our experiences. Therefore, we compared two different techniques:

1. Neurofilament Protein Clone 2F11 (NF, M 0762, Dako Ltd): In the first step, sections were incubated in 3% hydrogen peroxide for 10 min in order to inhibit the endogenous peroxides. After rinsing in distilled water, antibody retrieval was performed with steaming at 95 °C in pH 6 sodium citrate buffer. Sections were rinsed again and incubated with the NF antibodies (1:50) for 1 h at room temperature. Then, sections were rinsed again and incubated for 30 min at room temperature in an ImmPRESS™ anti-rabbit micropolymer (Vector Laboratories, Burlingame, CA) and developed with NovaRED™, a peroxidase substrate kit (Vector Laboratories, Burlingame, CA). After counterstaining with haematoxylin, sections were dehydrated and embedded in a Roti-Histokit II (Carl Roth, Karlsruhe, Germany). Controls of the immunohistochemical reactions were performed by using buffer instead of the primary antibody.
  Additionally to NF, we also used this protocol (modified in some steps) for calcitonin gene-related peptide (CGRP, ab81887, Abcam Ltd) and neuropeptide Y (NPY, ab30914, Abcam Ltd) as well as for cluster of differentiation 31 (CD31, sc-1506-R, Santa Cruz). Steaming was done at 95 °C using a pH of 9 (Dako Target Retrieval Solution). Incubation with antibodies (CD31 (1:50), CGRP (1:500), NPY (1:2000)) was also realized for 1 h at room temperature. The following steps were same as described for NF.
2. Neurofilament 200kD (NF-200, Abcam Ltd): The samples were embedded in paraffin. From every sample, 5-μm-thick sections were cut in a way that every sixth section was used for immunohistochemistry and all together five sections were stained from each sample. The sections were deparaffinised, rehydrated and cooked for 3 min in a pressure cooker in citrate buffer. After non-specific blocking in 5% aqueous solution of milk powder for 20 min, the sections were incubated with an antibody produced against neurofilament 200kD (NF-200, Abcam Ltd., UK, 1:400 overnight at room temperature [RT]). Then, the sections were treated with biotinylated anti-rabbit IgG (Vector Laboratories Inc., Burlingame, CA, USA, 1:400) followed by streptavidin-HRP incubation (Vector Laboratories Inc., Burlingame, CA, USA, 1:2000), each for 1 h at RT. The immunohistochemical reaction was visualized through the use of DAB (Sigma-Aldrich); some sections were counterstained with haematoxylin, and then, the sections were coverslipped.

### Quantification of NF-positive cells

**Fig. 1:**
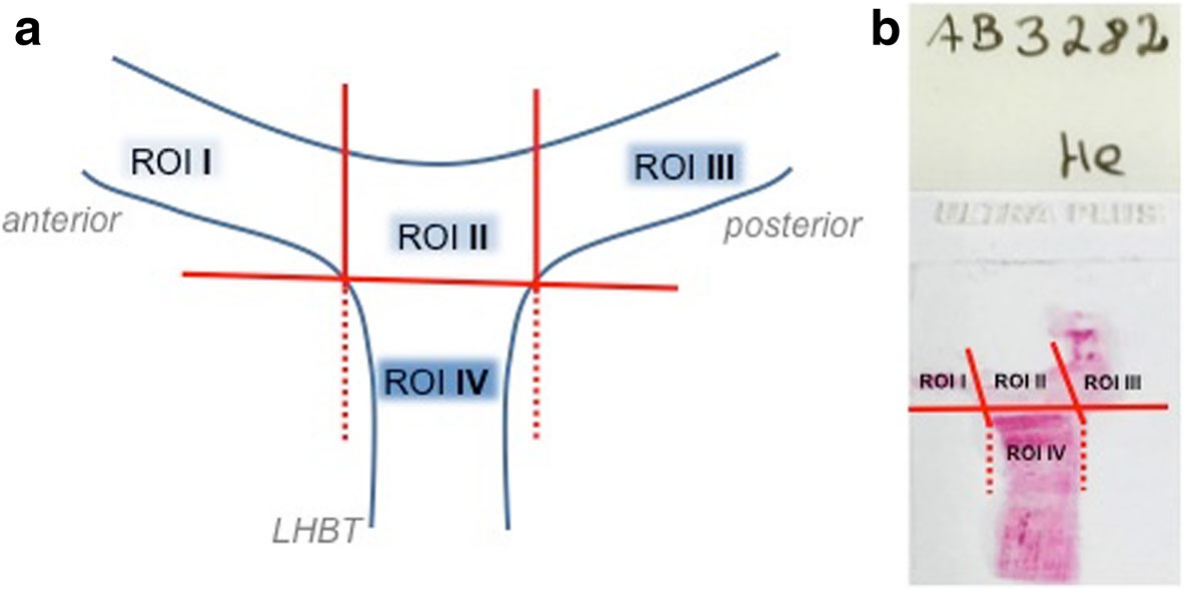
Definition of the regions of interest (ROIs) in the labral complex and the long head of the biceps tendon (LHBT). **a** ROI I represents the anterior superior aspect of the labral complex anterior to the LHBT origin. ROI II is the mid-portion of the superior labral complex at the LHBT origin (12 o’clock). ROI III represents the posterior superior aspect of the labral complex posterior to the LHBT origin, and ROI IV is the most proximal part of the LHBT before its attachment to the superior labrum. **b** Overview of a histological slide with the according ROIs

All slides were scanned at high resolution using a ×20 objective on a Dot-Slide scanning system (Olympus, Southend-on-Sea, UK). Thereafter, images were converted to tagged image file format (TIF). Regions of interest (ROI) were defined and accordingly drawn at all images using Photoshop CS5 (Adobe Systems Inc., San Jose, CA, USA). ROI included the following parts of the LHB tendon anchor and defined as follows (Fig. 1):

- ROI I: anterior superior labrum; anterior to the LHBT origin
- ROI II: mid-portion of the superior labrum at the origin of the LHBT (12 o’clock)
- ROI III: posterior superior labrum; posterior to the LHBT origin
- ROI IV: most proximal part of the LHBT before its attachment to the superior labrum

The entire TIF images were then automatically segmented according to the defined ROIs and measured using a programmed algorithm specifically created for this purpose in Definiens Developer XD 2.1 (Definiens AG, Munich, Germany).

The images were downscaled to 12.5% of the original size in order to detach the background from the tendon. The scanning region (entire tendon) was manually delineated. Tendon areas appearing white in the processed TIF images so far were not included in the further automated scanning and evaluation protocol. The images were then segmented using quadtree segmentation in which large squares are quartered until they fulfilled set homogeneity criteria. This results in large squares where the images are most homogenous, e.g. the background. The median brightness of the largest squares was measured to determine the approximate brightness of the background. The median brightness of all objects in the image was used to estimate the brightness of the tendon since a vast majority of objects are part of the tendon. The image objects were thresholded based on the measured values. Small regions enclosed by background were added to the background, and surface tension constrained region growing was performed to smooth the border between the tendon and the background. The resulting separation of background and tendon was synchronized to the full resolution image on which the segmentation of nerves was performed.

The parts of the images outside of the hand-drawn ROIs were excluded from further processing. The images were thresholded on the blue colour layer to identify parts of the image which may contain stained nerves. These candidate regions were expanded slightly and thresholded again based on their difference between the red and blue colour layers. The nerve segmentation was optimized using neighbourhood, size criteria and dilation/erosion.

**Fig. 2:**
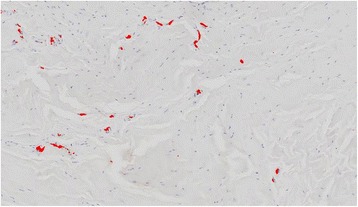
Depiction of the NF-positive-stained cells (NF-200, Abcam Ltd), represented by the red dots, as a result of the automated procedure using the Definiens protocol

The segmented nerves were counted, and their total size and the area of other tissue were measured separately for the different ROIs (Fig. 2).

### Statistical methods

All data obtained from the automated scanning process were then analysed by GraphPad Prism® (GraphPad Software Inc., Version 5.01). Kolmogorov-Smirnov test was applied to evaluate Gaussian data distribution. If data were distributed normally, a one-way analysis of variance was used to test for statistical significance between the different groups followed by post hoc Tukey’s multiple comparison test. If normality test revealed a non-Gaussian distribution, Kruskal-Wallis test followed by Dunn’s multiple comparison test was performed. A *p* value ≤ 0.05 was considered statistically significant. Furthermore, descriptive histological as well as immunohistochemical statistics were performed for all study groups.

## Results

**Fig. 3:**
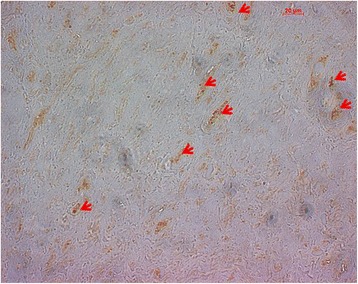
Example of an immunohistochemical slide at ROI I. Arrows indicating NF-positive-stained cells using Neurofilament 200kD (NF-200, Abcam Ltd)

In this study, we applied two different immunohistochemical staining methods of the LHBT labral complex against neurofilament. Interestingly, only one procedure (Neurofilament 200kD) resulted in qualitatively good neurofilament staining. The staining against CGRP and NPY failed in our specimen. An explanation could be that the examined tendons were presumably healthy and these neuropeptides are secreted primarily in neurogenic inflammation. These results underline the importance of selection of the immunohistochemical protocol, which should be applied in tendons (Fig. 3).

**Fig. 4:**
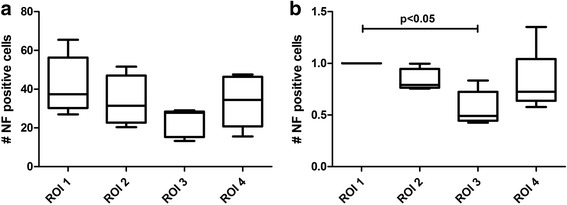
Quantitative measurement of NF-positive cells at the different ROIs based on the automated Definiens protocol. **a** Distribution of NF-positive cells in absolute numbers revealed a clear but insignificantly higher amount in favour of ROI I. **b** Setting ROI I at 100%, a significant difference could be seen compared to ROI III (ROI I vs. ROI III with a *p* value < 0.05)

Using the Neurofilament 200kD (NF-200, Abcam Ltd) antibody, a clear although not statistically significant distribution between the different ROIs could be found (Fig. 4a). ROI I, representing the anterior part of the LHBT labral anchor, showed the highest amount of NF-positive cells (42.1 ± 15) assessed by the automated detection and evaluation protocol. The density of NF-positive cells decreased the more posterior ROIs were measured (ROI II—33.7 ± 13.1; ROI III—23.1 ± 7.3). ROI IV being the most proximal part of the intraarticular LHBT following labral ROI II showed a similar amount of NF-positive cells as found in ROI II (ROI IV—33.8 ± 13.4). No age-related differences were found in the neurofilament distribution in the different ROIs (data not shown).

Nevertheless, setting the ROI I as 100%, it was found that a significantly lower number of NF-positive cells were present in ROI III compared to ROI I (*p* = 0.013), potentially explaining the persisting pain if type II SLAP lesions are addressed with suture anchors in their anterior aspect (Fig. 4b).

**Fig. 5:**
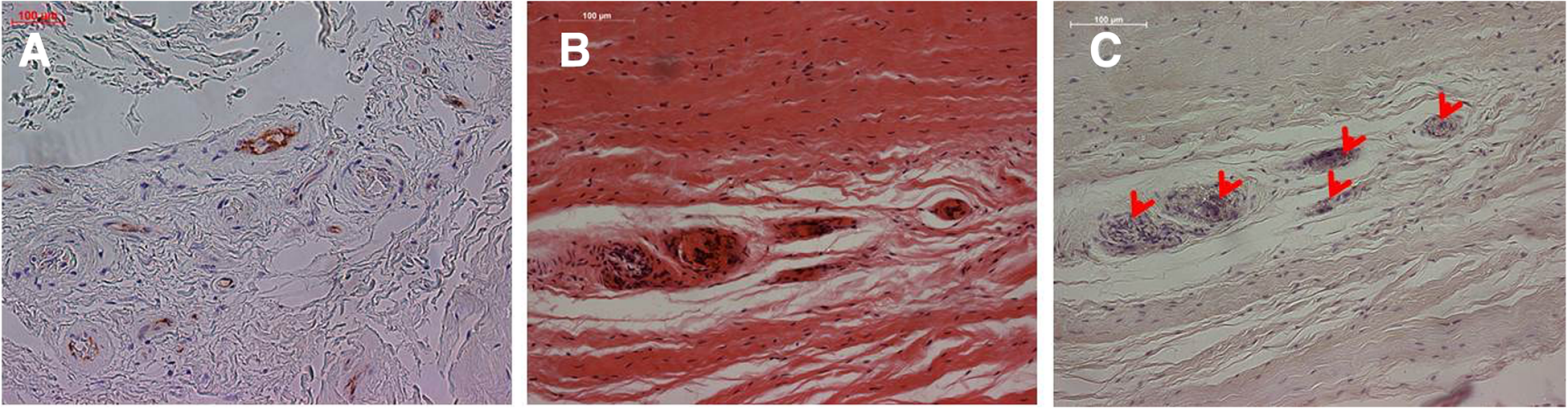
Representative immunohistochemical images depicting **a** CD31-positive cells, **b** H/E staining with vessels and **c** vessels accompanied with NF-positive cells (arrows)

Qualitative analysis showed that the NF-positive cells were primarily co-located to the vascular structures which were also found pre-dominantly in the anterior region = ROI I (Fig. 5). Interestingly, the attempt to stain these vascular structures against CD31 partly failed and we could not sufficiently depict vessels in order to apply the automated evaluation protocol. However, the standard H/E procedure was qualitatively adequate to recognize and analyse the vessels (Fig. 5).

**Fig. 6:**
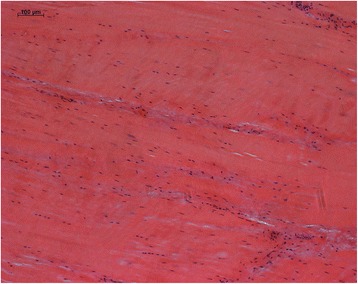
Representative H/E stained specimen showing qualitative characteristics of analysed tendons. Tendons appeared normal to only mildly degenerated without signs of acute or chronic inflammation

Assessing the variable parameter of the tendons, it was found that the specimen showed only mild degeneration patterns with flattened and spindle-shaped nuclei at a normal cellular amount (Fig. 6). Occasionally, nuclei were arranged moniliform without pathological substrate.

Similarly, collagen fibres were well organized in parallel without separation except sometimes due to alterations from the immunohistochemical specimen preparation. Undulating fibres in a waveform manner were also found from a mild to moderate degree.

In all analysed tendons, we could not find any signs of acute or chronic inflammation, which is normally appearing with higher cellularity and vascularity along with collagen fiber separation due to oedema.

## Discussion

The neurofilament antibody NF-200 used as general neuronal marker revealed the inhomogeneous distribution of neurofilament in the superior labrum with the highest density in the anterior labrum and the most proximal part of the LHBT. These results might give a reasonable explanation for the frequently observed fact that patients suffer from a prolonged period of pain after type II SLAP repair compared to LHBT-releasing procedures like tenodesis or tenotomy. To our knowledge, this is the first histological study investigating the neuronal density and distribution in the superior labrum, i.e. the SLAP region, additionally to the most proximal part of the LHBT.

In the literature, the LHBT has been discussed as pain generator in the glenohumeral joint either in tendinopathy [18], after proximal humeral fractures [19], or as a concomitant injured tendon in rotator-cuff tears [12, 17]. Several studies have been published that have investigated the nerve distribution in the shoulder joint capsule [14, 20], the subacromial bursa [21] or the coracoacromial ligament [22] immunohistochemically. Alpantaki et al. [15] was the first to study these neural elements in the LHBT. He found that the LHBT contains a large network of sensory and sympathetic nerve fibres, which is rather found predominantly near its insertion. These findings are well in line with our study results as we were able to show in the most proximal LHBT a nerve density comparable to the central or the proximal portion of the superior labrum. However, Alpantaki et al. [15] also stated that the type of innervation they found was not associated with blood vessels. This is in contrast to other studies as well as ours where nerve fibres were shown to accompany the surrounding blood vessels [23–25].

Tosounidis et al. [17] investigated the sympathetic innervation of the LHBT via immunohistochemical staining for protein S-100 and neuropeptide Y (NPY) and observed that the acute and chronic conditions of LHBT pathology share common morphological features and sympathetic innervation patterns. Thus, the authors suggest to perform either biceps tenotomy or tenodesis in hemiarthroplasties for proximal humeral fractures. However, these results might not be extrapolated on other isolated pathological conditions of the LHBT.

A histological and molecular analysis after tenotomy by Joseph et al. [16] revealed that the intra-articular portion of the LHBT displays many of the histological characteristics of tendinopathy whereas the extra-articular portion displays histological characteristics of a healthy tendon. These findings support our hypothesis that the pain generation in SLAP lesions derives from the most proximal part of the LHBT and even more from the superior labrum. Hadjileontis and Kontakis [26] even proved neuronal differentiation of tendon stromal cells as the source of biceps tendon pain, as these stromal cells are normally absent in normal tendons.

Summarizing previously performed studies, it seems to be evident that the LHBT acts as a pain generator with a higher density of neurofilament in the proximal parts and that inflammation leads to neural differentiation of tendon stromal cells inducing pain. Focusing on the pathology of the SLAP lesion, the injury itself might arise from a single trauma to the shoulder or from chronic repetitive microtraumata to the biceps tendon anchor as it is well known in overhead athletes. Thus, in the first case, the SLAP lesion has an acute onset whereas in the second case the genesis is more chronic with a possible concomitant inflammation. Independently of these injury mechanisms, literature is full of clinical studies dealing with the outcomes after surgical treatment of SLAP lesions [7–11, 27], in most cases referring to the type II lesion as it represents the most common type of injury [3].

Functional outcomes are described as satisfactory irrespective of the applied surgical technique [7, 8, 10, 11, 27]. However, some studies have shown that throwing athletes produce statistically inferior results than non-throwing athletes [28, 29]. High-demand athletes are likely to take a longer duration of rehabilitation in order to attain full recovery and—though in the minority of cases—the persisting pain after SLAP repair is a well known but still not fully understood condition [7–10, 27]. Interestingly, surgical techniques releasing the LHBT from the superior labrum (i.e. tenodesis or tenotomy) have shown to result in an immediate pain relief [10–12]. Probably, this is one of the reasons for the recently reported decrease of SLAP repairs performed [30, 31].

Taking a closer look at the surgical technique, the placement of the suture seems to play an important role. It is recommended to place the sutures for type II SLAP repair posteriorly of the LHBT in the superior glenoid. In case of an extended lesion reaching the anterior superior labrum, an additional anterior suture should be added [32]. By fixing the torn labrum to the already placed anchor in the glenoid, the labrum itself is either pierced or looped using a modified lasso-loop technique [6, 27], thus causing a strangling effect.

The results of our study show that the density of neurofilament is significantly higher in the anterior portion of the superior labrum independent of prior injury or pathology as the tendons and the proximal labrum were taken from macroscopically healthy tendons. During the healing process after SLAP repair, it might even come to higher differences as the inflammation process leads to an increased neuronal differentiation [26].

## Conclusions

Summarizing our results, the major finding of this study is the inhomogeneous distribution and density of neurofilament throughout the most proximal part of the LHBT and the superior labrum with the highest number of neurofilament in the anterior superior labrum. Thus, suture placement in type II SLAP repair might play an important role for the postoperative progress of pain relief. If possible, sutures should be placed only posterior to the LHBT insertion. However, the tear might reach the anterior portion of the superior labrum requiring an additional suture positioned anterior to the LHBT.

## Abbreviations

CD31: Cluster of differentiation 31
CGRP: Calcitonin gene-related peptide
LHBT: Long head of the biceps tendon
NF: Neurofilament
NPY: Neuropeptide Y
ROI: Region of interest
RT: Room temperature
SLAP: Superior labrum anterior to posterior
